# Responses to crizotinib and cabozantinib in patient with lung adenocarcinoma harboring mesenchymal-epithelial transition factor exon 14 skipping mutation: A case report

**DOI:** 10.1097/MD.0000000000024300

**Published:** 2021-01-29

**Authors:** Ruo-Yan Qin, Ling-Shuang Liu, Hui-Yong Zhang, Cheng-Hua Lu, Xiao-Yan Guo, Ling-Yue Zhang, Xin-Bei Yuan, Hong-Hao Xue

**Affiliations:** a Department of Oncology; b Department of Pulmonary Medicine, Longhua Hospital, Shanghai University of Traditional Chinese Medicine, Shanghai, China.

**Keywords:** cabozantinib, crizotinib, lung adenocarcinoma, mesenchymal-epithelial transition factor exon 14 skipping mutation

## Abstract

**Rationale:**

Lung cancer is a leading cause of cancer-related mortality worldwide. Currently, targeted therapy has proved highly efficient in the treatment of advanced non-small cell lung cancer (NSCLC). Mesenchymal-epithelial transition factor (MET) is considered a validated molecular target in NSCLC. Given the low incidence of MET exon 14 skipping mutation, the planning of precision treatment for patients is a clinical problem that needs to be solved. In this report, we present a *MET*-positive case that benefited from crizotinib and cabozantinib treatment.

**Patient concerns:**

A 77-year-old patient was diagnosed with lung adenocarcinoma in our hospital. Positron emission tomography-computed tomography (PET-CT) showed a right upper lobe mass (58 × 56 mm, SUVmax 15.6), right hilar enlarged lymph nodes, and multiple bone and left adrenal metastases (c-T3N1M1c).

**Diagnoses:**

*MET* exon 14 mutation (exon14, c.2888-1G>C) was examined using the lung puncture sample by next generation sequencing. Therefore, the patient was diagnosed with late-stage lung adenocarcinoma with MET exon14 skipping gene mutation.

**Interventions:**

Crizotinib was given as the first-line treatment from August 2019. Considering the resistance of crizotinib, cabozantinib was given for second-line treatment.

**Outcomes:**

Crizotinib was administered (250 mg bid) for 8 months, and her disease achieved partial regression (PR) and progression-free survival (PFS), which lasted for 8 months. The patient also reached PR after the second-line treatment with cabozantinib, and is currently under follow-up, with an overall survival (OS) of >12 months.

**Lessons:**

As *MET* exon 14 skipping mutation is rare in clinical practices, MET-TKIs (tyrosine kinase inhibitors) treatment can boost curative effects and improve prognosis of patients with advanced lung adenocarcinoma. This case report supports a rationale for the treatment of lung adenocarcinoma patients with a *MET* exon 14 skipping mutation and provides alternative treatment options for these types of NSCLC patients.

## 1 Introduction

Lung cancer is a malignant tumor with the highest mortality in the world, with a 5-year survival rate of only 15%. Non-small cell lung cancer (NSCLC) accounts for 85% of lung cancer. As patients with advanced NSCLC are deprived of the possibility of surgery, targeted therapies offer a chance for patients with driver gene alteration to prolong survival. Mesenchymal-epithelial transition factor (MET) has been in the spotlight in the recent years as a promising novel targeted therapy of NSCLC. Abnormal activation of the *MET* pathway can promote the proliferation, invasion, and migration of cancer cells, and eventually lead to the growth and development of malignant tumors,^[1]^ including *MET* 14 exon skipping mutation, gene amplification, and overexpression.^[2–4]^ The incidence of MET exon 14 skipping mutation stands at 3% to 6%^[5,6]^ in NSCLC, and 3% to 4%^[5,7]^ in lung adenocarcinoma. In pulmonary sarcomatoid carcinoma (PSC), a rare type of NSCLC, the mutation rate is as high as 31.8%.^[8]^ As an independent indicator of poor prognosis, *MET* exon 14 skipping mutation is more common in elderly, man, and advanced NSCLC patients,^[9–13]^ with various types and forms of mutation.^[14]^ MET-TKIs (tyrosine kinase inhibitors), such as crizotinib, capmatinib, and tepotinib were proved effective treatment for NSCLC patients from clinical trials. However, there is no clinical data of cabozantinib used in NSCLC patients with *MET* exon14 skipping mutation. In this report, we present a case with *MET* exon14 skipping mutation which benefited from cabozantinib treatment after the disease progressed of treatment with crizotinib in the real world.

## 2 Case presentation

**Figure 1:**
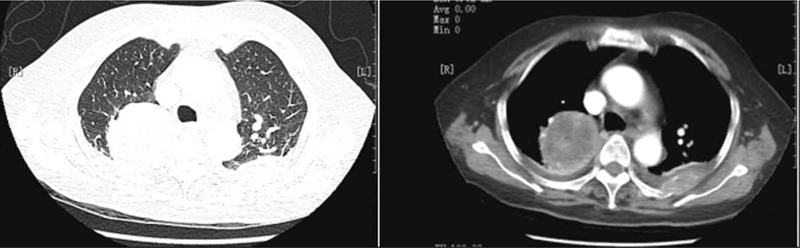
CT showed a mass in the upper lobe of the right lung, and the density of fluid on both sides (*left*: lung window, *right*: mediastinal window). CT = computed tomography.

**Figure 2:**
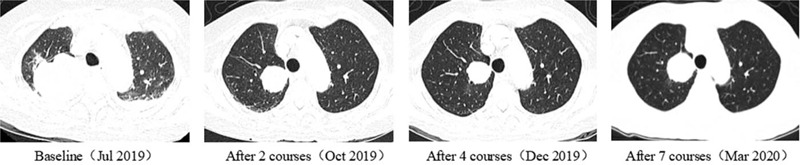
CT during the first-line treatment showed that the chest lesion was significantly reduced, and the patient reached PR. CT = computed tomography; PR = partial regression.

**Figure 3:**
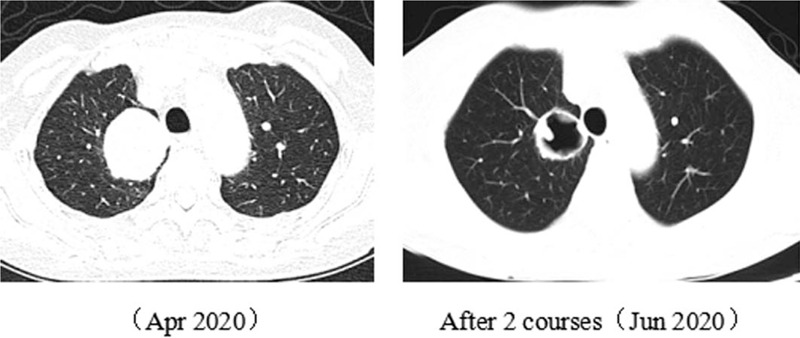
CT during the second-line treatment showed that the lesion was significantly reduced, cavities were formed, and the patient reached PR. CT = computed tomography; PR = partial regression.

**Figure 4:**
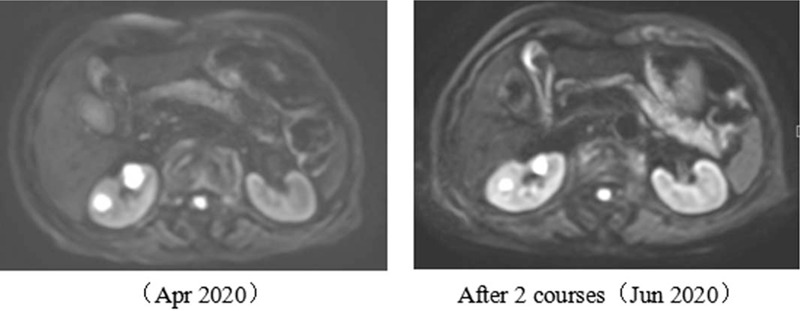
MRI showed the reduction of kidney metastases. MRI = magnetic resonance imaging.

A 77-year-old woman with a 1-month history of backache, fatigue, and weight loss was admitted. She had a history of deep vein thrombosis, and no other major medical problems. She underwent L2 vertebroplasty and was found with multiple lumbar metastases. Positron emission tomography-computed tomography (PET-CT) showed a right upper lobe mass (58 × 56 mm, SUVmax 15.6), right hilar enlarged lymph nodes and multiple bone, and left adrenal metastases (c-T3N1M1c) (Fig. 1). Lung puncture pathology suggested adenocarcinoma with *MET* exon 14 mutation (exon14, c.2888-1G>C) and *TP53* mutation (exon2,c.241C>T,p.R81X), PD-L1 tumor proportion score (TPS) 18.34%. As the patient had severe backache, she took 20 mg of OxyContin twice a day. The first-line targeted therapy was conducted with crizotinib (250 mg bid), and grade I diarrhea was observed. The patient reached partial regression (PR) after 2 courses of treatment. With symptomatic treatment such as ibandronate for bone protection and nutritional support, the symptoms of backache and fatigue were alleviated, allowing the patient to gradually reduce and stop the intake of OxyContin. On April 2, 2020, chest CT showed that the right upper lobe mass was larger than before while magnetic resonance imaging (MRI) identified multiple metastases in kidneys, suggesting progression disease (PD). The first-line treatment had a progression-free survival (PFS) of 8 months (Fig. 2). Considering the resistance of crizotinib, a second biopsy was conducted, indicating adenocarcinoma with MET exon 14 skipping mutation. During the second-line targeted therapy with cabozantinib (60 mg qd), she had grade II hand-foot syndrome, for which cabozantinib was adjusted to 40 mg qd. Later, only the adverse reaction of grade I hand-foot syndrome was observed, suggesting a significant improvement of life quality for the patient. Re-examination of chest CT after two courses of treatment detected a thick-walled cavity at the upper right lung (Fig. 3). MRI showed that the metastases of kidneys were smaller than before (Fig. 4). Overall evaluation of the efficacy was PR. The patient is still under follow-up, with an overall survival (OS) of >12 months.

## 3 Discussion

Chemotherapy is not an ideal treatment for NSCLC patients with *MET* exon 14 skipping mutation as the OS of patients receiving first-line chemotherapy is only 6.7 months.^[5]^ In terms of immunotherapy, a study of 147 NSCLC patients with *MET* exon 14 skipping mutations showed that: 63% of patients had positive expression of programmed death ligand 1 (PD-L1) (TPS ≥ 1%) while the level of tumor mutational burden (TMB) expression was generally low. Among 24 patients with *MET* exon 14 skipping mutation who received immunotherapy, the objective response rate (ORR) was 17%, and the median PFS was only 1.9 months.^[15]^

Targeted therapy is the first choice for the treatment of NSCLC patients with *MET* exon 14 skipping mutation, including multi-targeted drugs (crizotinib, cabozantinib) and selective targeted drugs (savolitinib, capmatinib, teponitinib). Crizotinib was originally a small molecule TKIs developed for *c-MET*. PROFILE 1001 study analyzed 18 patients with *MET* exon 14 skipping mutation, whose ORR reached 44% after treatment with crizotinib.^[16]^ A prospective study included 69 patients treated with crizotinib, whose ORR reached 32%, PFS reached 7.3 months, and duration of response (DoR) reached 9.1 months, with common adverse reactions such as grade I–II edema, visual impairment, and diarrhea.^[17]^ Cabozantinib is a multi-kinase inhibitor of *MET*, *VEGFR2*, *AXL*, *Tie2*, *KIT, FLT3*, and *RET*.^[18]^ Cabozantinib can not only serve as anti-tumor angiogenesis, but also combine with the inactive conformation of the ATP domain of the *MET* gene, with an inhibitory effect on the secondary mutation of *MET*. Common adverse reactions include diarrhea, fatigue, high blood pressure, hand-foot syndrome. Klempner reported a patient with liver, brain metastases, and *MET* exon 14 skipping mutation. First-line crizotinib induced remission of lung lesions, progression of intracranial lesions, and grade IV liver damage. In the second-line, the intracranial lesions disappeared with cabozantinib while the lung lesions continued to shrink, and liver transaminase returned to normal.^[19]^ It is suggested that cabozantinib may become a replacement of crizotinib in case of drug resistance. Recent researches show that *MET* inhibitors (savolitinib, capmatinib, teponitinib) have great advantages in efficacy. Savolitinib was used in the treatment of 41 NSCLC patients with *MET* exon 14 skipping mutation (PSC excluded), whose had an ORR of 48.8%, disease control rate (DCR) of 95.1%, and PFS of 9.7 months.^[13]^ The GEOMETRY mono-1 study showed that when capmatinib was used in the treatment of stage IIIb or stage IV NSCLC patients with *MET* exon 14 skipping mutation, there were 28 newly-treated patients who had an ORR of 67.9%, DCR of 96.4%, and PFS of 9.69 months, and there were 69 treated patients who had an ORR of 40.6%, DCR of 78.3%, and PFS of 5.42 months.^[20]^ The VISION study showed that patients with *MET* 14 exon skipping mutation, when treated with teponitinib, had an ORR of about 46.5%, DCR of 65.7%, and DoR of 11.1 months.^[21]^ With the deepening research of precision treatment, more patients will be able to achieve long-term survival.

This case demonstrates that targeted therapy has proved an ideal choice for patient suffering from advanced lung adenocarcinoma with *MET* exon 14 skipping mutation. The patient taking first-line treatment of crizotinib had significantly improved PR, and PFS of up to 8 months, consistent with previous studies. Meanwhile, symptoms like backache and fatigue were alleviated, analgesic was reduced and terminated, and her physical condition was improved. After the progression, the genetic test of the second biopsy reexamination still showed *MET* exon 14 skipping mutation, with the second-line treatment with cabozantinib as the first choice to improve the curative effects. Clinicians also observed the side effects and the overall tolerance of the patient. The toxicity of the targeted therapy in this case was generally tolerated, and with individualized symptomatic and supportive treatment, these factors were the key to the prolonged survival. *MET* 14 exon skipping mutation is a unique molecular subtype of NSCLC, featuring rarity and poor prognosis. Chemotherapy is not effective while immunotherapy needs further verification. In the era of precision treatment, targeted therapy brings breakthroughs and improves prognosis. However, limited availability of new drugs, individual differences in the types of *MET* exon 14 skipping mutations, and drug resistance are posing challenges to clinical practices, which requires further studies in the future.

## 4 Conclusion

For patients with *MET* exon 14 skipping mutation, MET-TKI can significantly improve the prognosis. The patient taking targeted therapy showed reduction of lung lesions, relief of the symptoms, improved quality of life, and prolonged survival.

## Author contributions

**Conceptualization:** Hong-Hao Xue, Ruo-Yan Qin, Xiao-Yan Guo.

**Data curation:** Cheng-Hua Lu.

**Formal analysis:** Hui-Yong Zhang.

**Investigation:** Ling-Yue Zhang.

**Methodology:** Ling-Shuang Liu, Hui-Yong Zhang, Ling-Yue Zhang.

**Project administration:** Hong-Hao Xue.

**Resources:** Cheng-Hua Lu, Xin-Bei Yuan.

**Software:** Xiao-Yan Guo.

**Validation:** Xiao-Yan Guo.

**Visualization:** Xin-Bei Yuan.

**Writing – original draft:** Ruo-Yan Qin.

**Writing – review & editing:** Hong-Hao Xue.

## Abbreviations

Abbreviations: DCR = disease control rate, DoR = duration of response, MET = mesenchymal-epithelial transition factor, NSCLC = non-small cell lung cancer, ORR = objective response rate, OS = overall survival, PD = progression disease, PD-L1 = programmed death ligand 1, PET-CT = positron emission tomography-computed tomography, PFS = progression-free survival, PR = partial regression, PSC = pulmonary sarcomatoid carcinoma, TKIs = tyrosine kinase inhibitors, TMB = tumor mutational burden.
